# Supplementary material for: Psychosocial impacts of COVID-19 in the Guinean population. An online cross-sectional survey
Source: PLoS One. 2021 Feb 2;16(2):e0245751. doi: 10.1371/journal.pone.0245751 (PMC7853443; doi:10.1371/journal.pone.0245751)
Supplement: S1 File — (DOCX) [file pone.0245751.s001.docx]

| Component 1 | | | | | | | |  |
| --- | --- | --- | --- | --- | --- | --- | --- | --- |
| **Row** | **Missings** | **Mean** | **SD** | **Skew** | **Item Difficulty** | **Item Discrimination** | **α if deleted** |  |
| Negative impact of COVID-19 | 14.18 % | 4.15 | 0.93 | -1.3 | 0.83 | 0.364 | 0.69 |  |
| Loss of job | 14.55 % | 3.59 | 1.1 | -0.42 | 0.72 | 0.457 | 0.667 |  |
| Not financial impact | 14.18 % | 1.89 | 1.08 | 1.28 | 0.38 | -0.164 | 0.807 |  |
| Hard time getting food | 14.18 % | 3.3 | 1.11 | -0.25 | 0.66 | 0.63 | 0.618 |  |
| Hard time getting means of transportation | 14.55 % | 3.64 | 1.17 | -0.66 | 0.73 | 0.604 | 0.623 |  |
| Hard time getting costing care | 14.18 % | 3.19 | 1.01 | -0.14 | 0.64 | 0.684 | 0.61 |  |
| Difficult to getting things needeed | 14.18 % | 3.91 | 1.04 | -1.21 | 0.78 | 0.489 | 0.659 |  |
| Mean inter-item-correlation=0.258 · Cronbach's α=0.710 | | | | | | | | |

| Component 1 | | | | | | | |  |
| --- | --- | --- | --- | --- | --- | --- | --- | --- |
| **Row** | **Missings** | **Mean** | **SD** | **Skew** | **Item Difficulty** | **Item Discrimination** | **α if deleted** |  |
| Giving money to support COVID-19 crisis | 14.18 % | 4.3 | 0.94 | -1.68 | 0.86 | 0.215 | 0.748 |  |
| support to government intiatives COVID-19 | 14.55 % | 4.21 | 0.81 | -1.43 | 0.84 | 0.637 | 0.55 |  |
| More.researches from.Guinea on OVID.19 | 14.55 % | 4.38 | 0.67 | -1.39 | 0.88 | 0.452 | 0.639 |  |
| Support to the government measures restrictions | 14.55 % | 4.21 | 0.81 | -1.43 | 0.84 | 0.637 | 0.55 |  |
| Need of strong government officials | 14.55 % | 4.33 | 0.82 | -1.75 | 0.87 | 0.359 | 0.674 |  |
| Mean inter-item-correlation=0.321 · Cronbach's α=0.688 | | | | | | | | |

| Component 1 | | | | | | | |  |
| --- | --- | --- | --- | --- | --- | --- | --- | --- |
| **Row** | **Missings** | **Mean** | **SD** | **Skew** | **Item Difficulty** | **Item Discrimination** | **α if deleted** |  |
| Punishing violation of wearing masks | 14.55 % | 4.2 | 0.93 | -1.28 | 0.84 | 0.629 | 0.767 |  |
| Punishing violation of curfew | 14.91 % | 4.06 | 0.9 | -0.99 | 0.81 | 0.631 | 0.766 |  |
| Punishing the gathering of more than 20 people | 14.55 % | 4.26 | 0.82 | -1.39 | 0.85 | 0.621 | 0.771 |  |
| Enforces social distancing | 14.55 % | 4.03 | 0.87 | -0.83 | 0.81 | 0.647 | 0.758 |  |
| Mean inter-item-correlation=0.522 · Cronbach's α=0.813 | | | | | | | | |
